# Ecchordosis Physaliphora: A Rare and Challenging Clinical Entity in a Patient With Acromegaly

**DOI:** 10.1210/jcemcr/luaf150

**Published:** 2025-08-29

**Authors:** Pedro Marques, Lia Neto, Francisco Tortosa, Amets Sagarribay

**Affiliations:** Pituitary Tumor Unit, Endocrinology Department, Hospital CUF Descobertas, 1998-018 Lisbon, Portugal; Faculty of Medicine, Universidade Católica Portuguesa, 1998-018 Lisbon, Portugal; Pituitary Tumor Unit, Radiology Department, Hospital CUF Descobertas, 1998-018 Lisbon, Portugal; Pituitary Tumor Unit, Pathology Department, Hospital CUF Descobertas, 1998-018 Lisbon, Portugal; Pituitary Tumor Unit, Neurosurgery Department, Hospital CUF Descobertas, 1998-018 Lisbon, Portugal

**Keywords:** pituitary adenoma, acromegaly, ecchordosis physaliphora, notochordal tumors, clival lesions

## Image Legend

**Figure 1.**
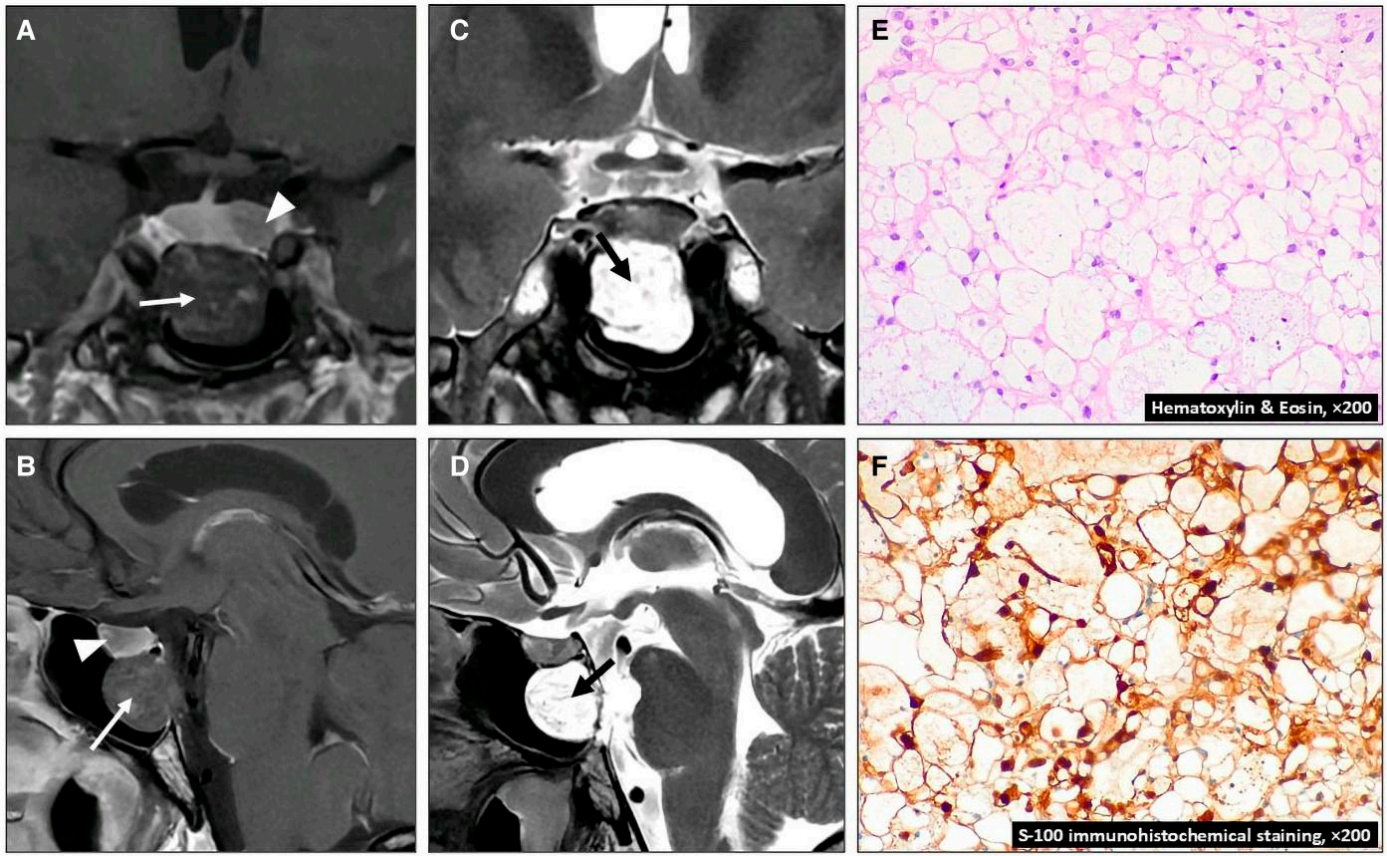
Appearance of the ecchordosis physaliphora on magnetic resonance imaging (A-D) and histopathological images (E and F).

A 38-year-old male was diagnosed with acromegaly based on clinical features and raised serum IGF-1 at 583 ng/mL (57-241) [76.2 nmol/L (7.5-31.5)]; serum prolactin was normal. Magnetic resonance imaging showed a T1 hypo-enhancing 10 mm pituitary adenoma (PA) (arrowheads), and a clival lesion (arrows) invading the sphenoid sinus with low-intensity and no gadolinium enhancement on T1 (Fig. 1A and 1B) and high-intensity on T2 (Fig. 1C and 1D), consistent with ecchordosis physaliphora (EP). Transsphenoidal hypophysectomy and clival biopsy were performed simultaneously. Histopathological analysis confirmed mammosomatotropinoma and EP. EPs are benign notochordal remnant lesions common at the retroclival prepontine cistern and are often incidentally discovered but may present with various symptoms, including headaches, rhinorrhea, and cranial nerve paralysis. EP poses significant diagnostic challenges with other clival lesions, notably chordomas [1]. Magnetic resonance imaging is the most useful diagnostic tool, as EP exhibits characteristic appearance: T1 hypointensity, T2 hyperintensity, and no gadolinium enhancement [1]. Histopathological analysis can help in establishing the EP diagnosis [1]. Typical features include physaliporous cells with large mucin-containing intracytoplasmic vacuoles (Fig. 1E) and S-100 immunopositivity (Fig. 1F), as we observed. EP has not been reported in PA patients, but intrasellar chordomas may coexist and/or mimic PAs [2]. This case alerts for the possibility of coincidental PAs and rare clival lesions and highlights the importance of neuroimaging and neuropathology for adequate diagnosis and management of EP.
